# MUSIC-Based Multi-Channel Forward-Scatter Radar Using OFDM Signals

**DOI:** 10.3390/s25247621

**Published:** 2025-12-16

**Authors:** Yihua Qin, Abdollah Ajorloo, Fabiola Colone

**Affiliations:** Department of Information, Electronics and Telecommunications Engineering (DIET), Sapienza University of Rome, 00184 Rome, Italy; abdollah.ajorloo@uniroma1.it (A.A.); fabiola.colone@uniroma1.it (F.C.)

**Keywords:** forward scatter radar, low-cost radar, MUSIC algorithm, OFDM signal, DoA estimation

## Abstract

This paper presents an advanced signal processing framework for multi-channel forward-scatter radar (MC-FSR) systems based on the Multiple Signal Classification (MUSIC) algorithm. The proposed framework addresses the inherent limitations of FFT-based space-domain processing, such as limited angular resolution and the poor detectability of weak or closely spaced targets, which become particularly severe in low-cost FSR systems, which are typically operated with small antenna arrays. The MUSIC algorithm is adapted to operate on real-valued data obtained from the non-coherent, amplitude-based MC-FSR approach by reformulating the steering vectors and adjusting the degrees of freedom (DoFs). While compatible with arbitrary transmitting waveforms, particular emphasis is placed on Orthogonal Frequency Division Multiplexing (OFDM) signals, which are widely used in modern communication systems such as Wi-Fi and cellular networks. An analysis of the OFDM waveform’s autocorrelation properties is provided to assess their impact on target detection, including strategies to mitigate rapid target signature decay using a sub-band approach and to manage signal correlation through spatial smoothing. Simulation results, including multi-target scenarios under constrained array configurations, demonstrate that the proposed MUSIC-based approach significantly enhances angular resolution and enables reliable discrimination of closely spaced targets even with a limited number of receiving channels. Experimental validation using an S-band MC-FSR prototype implemented with software-defined radios (SDRs) and commercial Wi-Fi antennas, involving cooperative targets like people and drones, further confirms the effectiveness and practicality of the proposed method for real-world applications. Overall, the proposed MUSIC-based MC-FSR framework exhibits strong potential for implementation in low-cost, hardware-constrained environments and is particularly suited for emerging Integrated Sensing and Communication (ISAC) systems.

## 1. Introduction

Forward-scatter radar (FSR) is a specialised bistatic radar configuration in which the transmitter (TX) and receiver (RX) are positioned such that the bistatic angle approaches 180°. When a target crosses the baseline between them, it disrupts the direct signal path, resulting in a distinctive amplitude variation in the received signal due to the shadowing effect. This effect is particularly prominent when the target is near the baseline, where the forward-scattered pattern causes enhanced power while demonstrating stability in both amplitude and phase. Consequently, the resulting amplitude modulation is only governed by the target instantaneous phase, enabling the detection of the target and estimation of its instantaneous bistatic Doppler along the trajectory. Although FSR systems are inherently limited in range and velocity resolution, they offer several practical advantages. These include effective detection of low radar cross-section (RCS) targets, robustness to stealth technology, and a simplified system architecture that facilitates low-cost solutions [1,2].

Additionally, FSR systems are well-suited to passive radar implementations, where the use of existing transmitters eliminates the need for an active source, thereby reducing system complexity due to enabling the deployment of a passive receiver alone. Further, it enables covert operation of the radar. This approach has been extensively explored using transmitters of opportunity, including Global Navigation Satellite Systems (GNSS) [3], Global System for Mobile Communications (GSM) [4], Long-Term Evolution (LTE) [5], radio and television broadcast signals [6,7], and Wi-Fi systems [8]. In this paper, we specifically focus on orthogonal frequency division multiplexing (OFDM) waveforms, which are ubiquitous across modern communication systems from Wi-Fi and cellular networks to digital television and radio broadcasting. This choice is particularly relevant in the context of Integrated Sensing and Communication (ISAC) [9,10], a rapidly emerging paradigm that envisions the convergence of radar sensing and wireless communications within a unified framework.

In conventional FSR systems, where a single antenna is employed, target detection is typically performed in the bistatic Doppler domain using time-domain processing, which analyses the amplitude modulation induced by targets as they move across the baseline [1,2,6,7].

Alternatively, a limited number of studies have investigated space-domain processing using a multi-channel FSR (MC-FSR), where detection is performed in the angular domain by analysing the amplitude modulation across spatially distributed antenna elements [11,12]. As the modulation rate varies with the target’s position, this approach enables effective estimation of the direction of arrival (DoA). In contrast to conventional array processing techniques, the proposed non-coherent approach avoids the need for phase synchronisation among antenna elements, while demonstrating robustness against phase noise. Furthermore, thanks to its implementation on individual time snapshots, it supports different waveform types and facilitates the suppression of direct-path signals across spatial samples by spatial filtering [12].

Due to the relatively small number of array elements compared to the abundance of time-domain samples, space-domain processing in MC-FSR faces inherent challenges in detecting weak targets and achieving fine angular resolution. These limitations become particularly critical in multi-target scenarios, where accurate angular discrimination is essential. The problem is further exacerbated by the aim of preserving low system complexity and cost, which typically restricts the number of antenna elements that can be deployed.

To address these challenges while maintaining system simplicity, in this paper we have explored the application of Multiple Signal Classification (MUSIC) algorithm, as a super-resolution technique, to the MC-FSR system. This study builds on the preliminary results presented in [13,14]. In particular, the MUSIC algorithm is adapted to operate directly on amplitude-only (real-valued) data and a comprehensive framework for the MUSIC-based MC-FSR using arbitrary waveforms is developed. Then, focusing on OFDM waveforms, we study their specific impact on target detection and DoA estimation performance of the proposed MUSIC-based approach. We address corresponding advantages and limitations and propose solutions based on sub-band approach and spatial smoothing.

In particular, the benefits of spatial smoothing are theoretically investigated, along with applicable trade-offs in terms of computational complexity and reduction in the available DoFs. Exploiting the sub-band approach proposed in [6], we can address the decay of moving target signatures resulting from the autocorrelation properties of OFDM signals, though it introduces a trade-off by reducing the MUSIC response of stationary (or slowly moving) targets. The sub-band technique and the spatial smoothing can be combined, as needed, to make the system robust across a range of target dynamics.

The benefits and limitations of the MUSIC-based MC-FSR approach are explored through detailed discussion and simulation, offering practical insights into its deployment. The proposed approach shows significant improvement in angular resolution, enabling reliable discrimination of closely spaced targets, despite using a limited number of channels on receipt.

In addition, a series of dedicated experimental tests using OFDM signals are conducted to assess real-world feasibility. For this purpose, an S-band multi-channel FSR system was implemented using software-defined radios (SDRs) and commercial Wi-Fi antennas. The experimental setups include scenarios involving cooperative targets such as people and drones moving across different trajectories. The results confirm the effectiveness and practicality of the proposed approach under realistic operating conditions.

The structure of the paper is as follows: Section 2 summarizes the MC-FSR signal model and the space-domain processing principle. The MUSIC-based MC-FSR processing scheme is introduced in Section 3. A theoretical analysis of the effect of OFDM waveform is given in Section 4. Section 5 presents the simulation results. Section 6 describes the experimental setup and reports corresponding results to demonstrate the effectiveness of the proposed approach under real-world conditions. Finally, conclusions are drawn in Section 7.

## 2. MC-FSR Signal Model and Space-Domain Processing Principle

**Figure 1:**
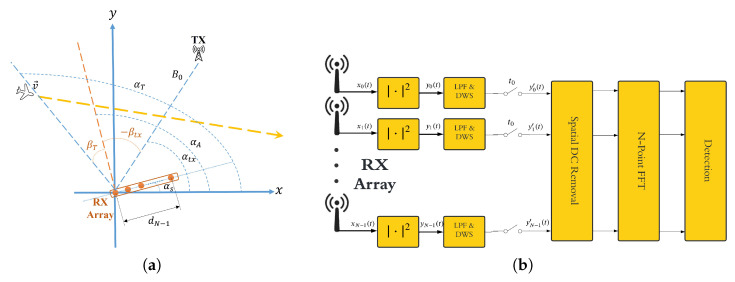
(**a**) MC-FSR geometry; (**b**) FFT-based space-domain signal processing scheme for MC-FSR.

Figure 1 shows the geometry and the signal processing block-diagram of an MC-FSR system. The receiver (RX) consists of a linear array of *N* identical antennas, with the first element, designated as the reference, positioned at the origin of a Cartesian coordinate system. The distance between the *n*-th element and the origin is denoted by $d_{n}$, where n=0,…,N−1, and $d_{0}=0$. The entire RX array is oriented at an angle $\alpha_{s}$ counterclockwise from the positive x-axis, resulting in a broadside angle of $\alpha_{A}=\alpha_{s}+\frac{\pi }{2}$. The transmitter (TX) is placed at a distance $B_{0}$ from the origin and forms an angle $\alpha_{tx}$ with respect to the positive x-axis. The TX transmits a waveform denoted by s(t) in the baseband.

In the absence of a target, the received signals at the RX array are exclusively due to the direct path from the transmitter, along with additive receiver noise. Assuming standard far-field and narrow-band conditions, the signal received at the *n*-th antenna can be modeled as

(1) $$x_{n}\left(t\right)=A_{D}s\left(t\right)e^{j\phi_{D,n}}+z_{n}\left(t\right),\;n=0,\ldots ,N-1,$$

where the coefficient $A_{D}=\frac{a}{B_{0}}e^{-j\frac{2\pi }{\lambda }B_{0}}$ encapsulates the free-space path loss and phase delay between the transmitter and the reference RX element (i.e., n=0). The phase shift at the *n*-th receiver due to array geometry is given by

(2) $$\phi_{D,n}=-\frac{2\pi }{\lambda }d_{n}\sin\left(\beta_{tx}\right),$$

with $\beta_{tx}=\alpha_{tx}-\alpha_{A}$. The noise component $z_{n}\left(t\right)$ represents additive white Gaussian noise (AWGN), modeled as a zero-mean circularly symmetric complex Gaussian variable.

As in conventional single-channel FSR systems [2,6,15,16], the squared amplitude of the received signals are exploited in the proposed MC-FSR system:

(3) $$\begin{array}{cc} y_{n}\left(t\right) & ={\left|A_{D}s\left(t\right)\right|}^{2}+{\left|z_{n}\left(t\right)\right|}^{2}+2ℜ\left\{A_{D}s\left(t\right)e^{j\phi_{D,n}}\zeta_{n}^{*}\left(t\right)\right\}, \end{array}$$

where ℜ{·} denotes the real part. It is straightforward to observe that, aside from the noise terms, the received signal maintains a constant amplitude across all array elements at a given time. Consequently, when the direct signal-to-noise ratio (DNR) is sufficiently high, a nearly uniform amplitude profile is obtained across the array, independent of the transmitted waveform.

Now, let us consider the case where a target crosses the baseline, forming an angle $\alpha_{T}\left(t\right)$ with respect to the positive x-axis. Under this condition, the received signal at the *n*-th element becomes

(4) $$\begin{array}{cc} x_{n}\left(t\right) & =A_{D}s\left(t\right)e^{j\phi_{D,n}}\\ \; & +A_{T}\left(t\right)s\left(t-\tau_{T}\left(t\right)\right)e^{j\phi_{T,n}\left(t\right)}\\ \; & +z_{n}\left(t\right),\;n=0,\ldots ,N-1. \end{array}$$

The second term in (4) represents the signal scattered by the target in the forward direction. There, the time-varying complex amplitude $A_{T}\left(t\right)=\varepsilon \left(t\right)A_{D}$ models the overall TX-target-RX path loss as well as the target’s forward-scattering characteristics (see [2] for the detailed formulations). Further,

(5) $$\tau_{T}\left(t\right)=\frac{1}{c}\left[R_{tx}\left(t\right)+R_{rx,0}\left(t\right)-B_{0}\right],$$

is the (bistatic) delay of target-scattered signal in which $R_{tx}\left(t\right)$ and $R_{rx,0}\left(t\right)$ represent the target distance from the transmitter and the reference receiver, respectively; it quantifies the excess path length relative to the direct signal. The corresponding instantaneous phase at the *n*-th array element is given by

(6) $$\begin{array}{cc} \phi_{T,n}\left(t\right) & =-2\pi f_{c}\tau_{T}\left(t\right)-\frac{2\pi }{\lambda }d_{n}\sin\left(\beta_{T}\left(t\right)\right) \end{array}$$

where $\beta_{T}\left(t\right)=\alpha_{T}\left(t\right)-\alpha_{A}$ denotes the angle of arrival of the target signal relative to the array broadside.

The expression for the squared signal amplitude in target presence becomes

(7) $$\begin{array}{cc} y_{n}\left(t\right) & ={\left|A_{D}s\left(t\right)\right|}^{2}+{\left|A_{T}\left(t\right)s\left(t-\tau_{T}\left(t\right)\right)\right|}^{2}+{\left|z_{n}\left(t\right)\right|}^{2}\\ \; & +2ℜ\left\{A_{D}s\left(t\right)A_{T}^{*}\left(t\right)s^{*}\left(t-\tau_{T}\left(t\right)\right)e^{j\left[\phi_{D,n}-\phi_{T,n}\left(t\right)\right]}\right\}\\ \; & +2ℜ\left\{A_{D}s\left(t\right)e^{j\phi_{D,n}}z_{n}^{*}\left(t\right)\right\}\\ \; & +2ℜ\left\{A_{T}\left(t\right)s\left(t-\tau_{T}\left(t\right)\right)e^{j\phi_{T,n}\left(t\right)}z_{n}^{*}\left(t\right)\right\}. \end{array}$$

Under typical conditions of high DNR and $A_{T}\ll A_{D}$, we get

(8) $$\begin{array}{cc} y_{n}\left(t\right) & \approx {\left|A_{D}s\left(t\right)\right|}^{2}\\ \; & +2ℜ\left\{A_{D}s\left(t\right)A_{T}^{*}\left(t\right)s^{*}\left(t-\tau_{T}\left(t\right)\right)e^{j\left[\phi_{D,n}-\phi_{T,n}\left(t\right)\right]}\right\}+\zeta_{n}\left(t\right) \end{array}$$

where $\zeta_{n}\left(t\right)=2ℜ\left\{A_{D}s\left(t\right)e^{j\phi_{D,n}}z_{n}^{*}\left(t\right)\right\}$ represents the observation noise in the signal model and is a real-valued Gaussian random process. Substituting from (2) and (6), we further simplify the signal model in (8) as

(9) $$\begin{array}{cc} y_{n}\left(t\right) & ={\tilde{A}}_{D}\left(t\right)\\ \; & +{\tilde{A}}_{T}\left(t\right)\cos\left(\frac{2\pi }{\lambda }d_{n}\underset{u(t)}{\underset{︸}{\left(\sin\beta_{T}\left(t\right)-\sin\beta_{tx}\right)}}+\;\phi \left(t\right)\right)+\zeta_{n}\left(t\right) \end{array}$$

where ${\tilde{A}}_{D}\left(t\right)≜{\left|A_{D}s\left(t\right)\right|}^{2}$, ${\tilde{A}}_{T}\left(t\right)≜2\left|A_{D}s\left(t\right)\right|\left|A_{T}\left(t\right)s\left(t-\tau \left(t\right)\right)\right|$, and $\phi \left(t\right)=\frac{2\pi }{\lambda }\left[R_{tx}\left(t\right)+R_{rx,0}\left(t\right)-B_{0}\right]+∠\left[A_{D}s\left(t\right)A_{T}^{*}\left(t\right)s^{*}\left(t-\tau \left(t\right)\right)\right]$. Even if we assume a constant-amplitude waveform, it is clear that the second term in (9) introduces amplitude modulation which is a sole effect of the presence of the target. The temporal amplitude variation, which is governed by the instantaneous phase term ϕ(t), is the primary feature exploited by time-domain processing in conventional single-channel FSR for target detection in the bistatic Doppler domain. However, according to the formula, amplitude also varies spatially across array elements (i.e., with *n*), which is the key point in the principle of operation of a MC-FCR [11,12]. Specifically, the rate of spatial amplitude modulation, with respect to the antenna index *n*, is governed by the DoA parameter $u\left(t\right)=\sin\beta_{T}\left(t\right)-\sin\beta_{tx}$. In order to exploit this effect, the signal processing scheme in Figure 1b can be adopted. Specifically, a spatial DC removal is first performed by subtracting the DC component of the signal estimated across the rx channels at each time snapshot. For sufficiently large *N*, this stage is expected to cancel out the first term in (8) as it does not depend on spatial index *n*. Therefore, for a specific time snapshot at $t=t_{0}$, we obtain

(10) $$w_{n}\left(t_{0}\right)={\tilde{A}}_{T}\left(t_{0}\right)\cos\left(\frac{2\pi }{\lambda }d_{n}u\left(t_{0}\right)+\phi \left(t_{0}\right)\right)+\zeta_{n}\left(t_{0}\right).$$

Consequently, conducting a spatial frequency analysis on individual signal snapshots holds the potential to detect the presence of a target and subsequently estimate its DoA. In the block diagram shown in Figure 1b, this spatial frequency analysis is performed using a fast Fourier transform (FFT), under the assumption that a uniform linear array (ULA) is employed. The resulting FFT output is then used to perform threshold-based detection in the DoA domain.

It is worth mentioning that although this space-domain processing for MC-FSR may appear similar to conventional phased-array techniques, it fundamentally differs in that target detection and localization are performed using an amplitude-based, inherently non-coherent approach. As a result, owing to its distinct characteristics compared to traditional phased array processing—such as much simpler array calibration—it achieves a notable reduction in system complexity, albeit with some inherent limitations, including sign ambiguity [12].

Whilst the FFT-based space-domain processing described above provides a straightforward solution for MC-FSR, it suffers from notable limitations. In particular, unlike the FFT-based time-domain FSR processing, which benefits from a large number of available time samples, the detection performance and the angular resolution are inherently constrained by the number of array elements, making it difficult to distinguish closely spaced or weak targets. Taking into account the primary purpose of having a low-cost system, this becomes especially problematic in multi-target scenarios under strict hardware constraints where only a limited number of antennas are deployed. In the next section, we seek application and adaptation of the MUSIC algorithm, as a super-resolution technique, to address this important challenge.

## 3. MUSIC-Based MC-FSR Processing Framework

To overcome the limitations in angular resolution and ambiguity handling associated with FFT-based space-domain processing, this section introduces the application of the MUSIC algorithm to the MC-FSR framework. MUSIC is a well-established super-resolution technique for DoA estimation in phased array systems. It exploits the eigenspace structure of the covariance matrix to distinguish between signal and noise subspaces, thereby achieving superior angular discrimination. However, its direct application to MC-FSR requires specific adaptations. In particular, since MC-FSR operates on amplitude-only, real-valued signal snapshots, the conventional complex-valued steering vectors must be reformulated, and the number of DoFs appropriately adjusted. These modifications are essential for maintaining accurate subspace separation and ensuring the algorithm’s effectiveness under real-valued conditions.

We let the RX array to be a ULA ($d_{n}=nd$). Then, considering a multi-target scenario with *k* targets, the received signal model in (9) (before DC removal) can be rewritten as:

(11) $$\begin{array}{cc} y_{n}\left(t\right) & ={\tilde{A}}_{D}\left(t\right)+\sum_{k=1}^{K}{\tilde{A}}_{T,k}\left(t\right)[\cos\phi_{k}\left(t\right)\cos\left(\frac{2\pi }{\lambda }ndu_{k}\left(t\right)\right)\\ \; & \;-\sin\phi_{k}\left(t\right)\sin\left(\frac{2\pi }{\lambda }ndu_{k}\left(t\right)\right)]+\zeta_{n}\left(t\right), \end{array}$$

where ${\tilde{A}}_{T,k}\left(t\right)$, $\phi_{k}\left(t\right)$, and $u_{k}\left(t\right)$ are defined as in (9) for the *k*-th target (k=1,…,K). The matrix form of the received signal is given by:

(12) y(t)=Bα(t)+z(t)

where $y\left(t\right)={\left[y_{0}\left(t\right),\ldots ,y_{N-1}\left(t\right)\right]}^{T}$ is the received signal vector, and $z\left(t\right)={\left[\zeta_{0}\left(t\right),\ldots ,\zeta_{N-1}\left(t\right)\right]}^{T}$ is the noise vector, which is a zero-mean white Gaussian vector with covariance matrix $E\left[zz^{T}\right]=\sigma^{2}I_{N}$. The amplitude α(t) is a 2K×1 vector, defined as:

$$\begin{array}{cc} \alpha (t)=\; & \left[{\tilde{A}}_{D}\left(t\right)\left[1,0\right],\;{\tilde{A}}_{T,1}\left(t\right)\left[\cos\phi_{1}\left(t\right),\;\sin\phi_{1}\left(t\right)\right],\right.\\ \; & \;{\left.\ldots ,\;{\tilde{A}}_{T,K}\left(t\right)\left[\cos\phi_{K}\left(t\right),\;\sin\phi_{K}\left(t\right)\right]\right]}^{T}, \end{array}$$

and the N×2K matrix B is given by:

$$B=\left[A\left(u_{0}\right)\;\;A\left(u_{1}\right)\;\;\ldots \;\;A\left(u_{K}\right)\right],$$

where each steering matrix A(u) is defined as:

(13) $$A\left(u\right)=\left[\begin{array}{cc} \cos\left(\frac{2\pi d}{\lambda }\cdot 0\cdot u\right) & -\sin\left(\frac{2\pi d}{\lambda }\cdot 0\cdot u\right)\\ \vdots & \vdots \\ \cos\left(\frac{2\pi d}{\lambda }\cdot \left(N-1\right)\cdot u\right) & -\sin\left(\frac{2\pi d}{\lambda }\cdot \left(N-1\right)\cdot u\right) \end{array}\right].$$

Here, A(u) is the steering matrix at direction *u*, and $u_{0}=0$ corresponds to the direct-path signal.

It is important to emphasize that the classical MUSIC algorithm—formulated for complex-valued, phase-coherent array measurements—cannot be directly applied to the MC-FSR system. In MC-FSR, the receiver operates on real-valued amplitude-only snapshots obtained after squaring the received signal magnitude. As a result, the standard complex steering vectors used in coherent array processing do not represent the physical measurement model of amplitude-based MC-FSR. Furthermore, the direct-path signal always contributes an additional deterministic component that must be explicitly treated as a separate source. For these reasons, the conventional MUSIC formulation would lead to an incorrect signal subspace structure, an incorrect dimensionality of the subspace, and ultimately an inconsistent DoA estimate. These fundamental differences necessitate a dedicated adaptation of the MUSIC algorithm to operate correctly in amplitude-only, non-coherent MC-FSR settings.

To extract the signal and noise subspaces, an eigenvalue decomposition of the sample covariance matrix $R_{y}=\frac{1}{M}\sum_{m=1}^{M}y\left(t_{m}\right)y^{T}\left(t_{m}\right)$ is performed:

(14) $$R_{y}=\left[\begin{array}{cc} E_{s} & E_{z} \end{array}\right]\left[\begin{array}{cc} \Sigma_{s} & 0\\ 0 & \Sigma_{z} \end{array}\right]\left[\begin{array}{c} E_{s}^{T}\\ E_{z}^{T} \end{array}\right]=\sum_{k=1}^{N}\lambda_{k}e_{k}e_{k}^{T}.$$

The signal subspace, denoted by $E_{s}=\left[e_{1},\ldots ,e_{p}\right]$, comprises the eigenvectors associated with the *p* largest eigenvalues of $R_{y}$, corresponding to the dominant signal components. The noise subspace, given by $E_{z}=\left[e_{p+1},\ldots ,e_{N}\right]$, consists of the remaining N−p eigenvectors associated with the smaller eigenvalues. In the context of MC-FSR with *K* signal sources, the signal subspace must include p=2K+1 eigenvectors. This accounts for *K* real-valued target signals and the contribution from the direct-path signal.

The peaks in the MUSIC spatial spectrum, evaluated over a specified range of *u*, correspond to the DoA of the targets. Using the estimated noise subspace $E_{z}$ and the steering matrix A(u), the MUSIC spatial spectrum is computed as:

(15) $$P\left(u\right)=\frac{1}{\mathrm{tr}\;\left[A^{T}\left(u\right)E_{z}E_{z}^{T}A\left(u\right)\right]}.$$

**Figure 2:**
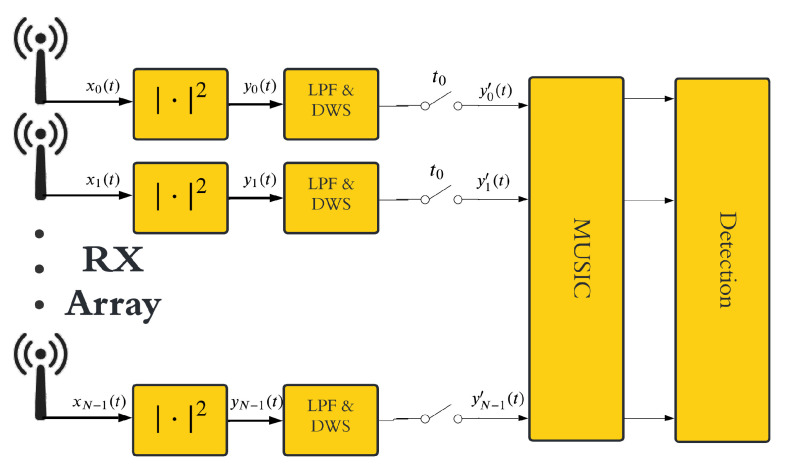
MUSIC-based MC-FSR signal processing scheme.

Since the MUSIC algorithm allows discriminating all present sources, including the direct-path signal from the transmitter, a dedicated DC removal stage becomes unnecessary. As a result, the overall signal processing scheme is streamlined as in Figure 2.

A main challenge associated with MUSIC algorithm is the processing of the correlated signals which is typical in radar scenarios. In this regard, spatial smoothing is a well-established technique for decorrelating signals. This is achieved by partitioning the full array into *L* overlapping subarrays, each consisting of N−L+1 elements, where L<N and N−L+1>p, with *p* denoting the number of DoFs. The smoothed covariance matrix is obtained by averaging the covariance matrices computed for each subarray $R_{s}=\frac{1}{L}\sum_{l=1}^{L}R_{\mathrm{sub},l}$. This matrix $R_{s}$ is then used in place of the original covariance matrix within the MUSIC algorithm to mitigate the degrading effects of source correlation.

The effectiveness of spatial smoothing strongly depends on the choice of the number of subarrays. When the number of antennas is limited, this choice is highly constrained, as it may leads to the lack of required DoFs, degrading the system performance. In addition, spatial smoothing introduces extra computational complexity, which may be unjustified in cases where signal correlation is not critical.

## 4. Effect of OFDM Waveforms and Sub-Band Processing

Applying the MUSIC algorithm to MC-FSR using different waveforms can lead to different results, as the output is inherently influenced by the specific characteristics of the adopted signals, especially in terms of signal autocorrelation. In this context, incorporating spatial smoothing may offer certain advantages, but it can also introduce potential drawbacks. In the following, we specifically investigate the impact of using an OFDM waveform to enable efficient and targeted use of spatial smoothing. This ensures improved system performance while maintaining computational efficiency and cost-effectiveness.

To this purpose, we focus on a single-target scenario and rewrite the noiseless received signal model in (11) as:

(16) $$\begin{array}{cc} y_{n}\left(t\right) & =\xi_{0}\left(t\right)e^{j\frac{2\pi }{\lambda }ndu_{0}}\\ \; & \;+\xi_{1}\left(t\right)e^{j\frac{2\pi }{\lambda }ndu_{1}\left(t\right)}+\xi_{1}^{*}\left(t\right)e^{-j\frac{2\pi }{\lambda }ndu_{1}\left(t\right)}, \end{array}$$

where $u_{0}=0$, and

(17) $$\begin{array}{cc} \xi_{0}\left(t\right) & ={\left|A_{D}\right|}^{2}s\left(t\right)s^{*}\left(t\right),\\ \xi_{1}\left(t\right) & =A_{D}A_{T}^{*}\left(t\right)s\left(t\right)s^{*}\left(t-\tau \left(t\right)\right)e^{j\phi (t)} \end{array}$$

denote the direct and target-path signal components, respectively. For simplicity, the subscript indices in $A_{T}$, τ, and ϕ are omitted.

We assume that the transmitted signal s(t) is a wide-sense stationary (WSS) random process, independent of ϕ(t). Our analysis focuses on the statistical properties of the received signals over short time intervals, which are typically used for covariance matrix estimation. Within such intervals, $A_{T}\left(t\right)$ and τ(t) are treated as time-invariant, so that we omit the time dependence. In contrast, ϕ(t), which varies more rapidly, remains time-dependent. The correlation between the direct-path signal $\xi_{0}\left(t\right)$ and the target-path signal $\xi_{1}\left(t\right)$ can thus be expressed as:

(18) $$\begin{array}{cc} \; & E\left[\xi_{0}\left(t\right)\xi_{1}^{*}\left(t\right)\right]=\\ \; & \;\;\varepsilon {\left|A_{D}\right|}^{4}\;E\left[s\left(t\right)s^{*}\left(t\right)s^{*}\left(t\right)s\left(t-\tau \right)\right]\cdot E\left[e^{-j\phi (t)}\right] \end{array}$$

where we have used $A_{T}=\varepsilon A_{D}$. As is apparent, the correlation between the two signal sources depends both on the waveform characteristics, via the second factor in the r.h.s. of (18), and on the motion of the target through the last factor in the r.h.s. of (18). For an OFDM signal with a sufficiently large number of subcarriers, the central limit theorem (CLT) enables us to approximate s(t) as a complex Gaussian process. As a result, s(t), $s^{*}\left(t\right)$, and $s^{*}\left(t-\tau \right)$ can all be regarded as circularly symmetric complex Gaussian random variables. Under these conditions, by expanding the expectation term in (18), we have

(19) $$\begin{array}{cc} E\; & \left[s\left(t\right)s^{*}\left(t\right)s^{*}\left(t\right)s\left(t-\tau \right)\right]=\\ \; & \;2E\;\left[s\left(t\right)s^{*}\left(t\right)\right]E\;\left[s^{*}\left(t\right)s\left(t-\tau \right)\right]+\\ \; & \;E\;\left[s(t)s(t-\tau )\right]E\;\left[s^{*}\left(t\right)s^{*}\left(t\right)\right]. \end{array}$$

Since for a circularly symmetric complex Gaussian variable *X*, we have $E\left[X^{2}\right]=0$, the second term vanishes. Consequently, (18) reduces to

(20) $$E\left[\xi_{0}\left(t\right)\xi_{1}^{*}\left(t\right)\right]=2\varepsilon {\left|A_{D}\right|}^{4}\;\sigma_{s}^{2}R_{s}\left(\tau \right)\cdot E\left[e^{-j\phi (t)}\right],$$

where $\sigma_{s}^{2}=E\left[{\left|s(t)\right|}^{2}\right]$ is the signal power and $R_{s}\left(\tau \right)=E\left[s^{*}\left(t\right)s\left(t-\tau \right)\right]$ denotes the autocorrelation function of s(t).

For a moving target, the time-varying phase ϕ(t), caused by the Doppler effect, significantly reduces the correlation between the target-path signal $\xi_{1}$ and the direct-path signal $\xi_{0}$, irrespective of the signal auto-correlation $R_{s}\left(\tau \right)$. In particular, if the target’s velocity is sufficiently high, the resulting Doppler shift introduces rapid phase variations, effectively decorrelating the signals across snapshots. In the limiting case, ϕ(t) can be modeled as a white random process uniformly distributed over [0,2π]. Consequently, $E\left[e^{-j\phi (t)}\right]=0$, implying that $\xi_{0}$ and $\xi_{1}$ become completely uncorrelated. Under such conditions, spatial smoothing is no longer necessary.

However, when the target is stationary, the phase term is time-invariant (i.e., zero Doppler), so $\phi \left(t\right)=\phi_{0}$ is constant. Consequently, $E\left[e^{-j\phi (t)}\right]$ is also a constant, which can be absorbed into ε. This means, even for a stationary target or one with a small bistatic Doppler shift, the correlation between $\xi_{0}$ and $\xi_{1}$ decreases since it directly reflects the correlation properties of the originals signal s(t).

The autocorrelation function of an OFDM signal can be expressed by $R_{s}\left(\tau \right)=\mathrm{sinc}\left(B\tau \right)$, which is inversely proportional to the waveform bandwidth *B* and the delay τ. As the bistatic delay increases, the correlation diminishes, which is advantageous for MUSIC-based processing. On the other hand, this reduction in correlation adversely affects the fundamental FSR principle, as it decreases the average power of the target-path signal $\xi_{1}\left(t\right)$[6]. In particular, it has been shown that the expected output target signature in space-domain processing is proportional to $R_{s}\left(\tau \right)$[12]. Consequently, due to the large bandwidth and corresponding sinc-shaped $R_{s}\left(\tau \right)$ of OFDM waveforms, target response becomes susceptible to rapid fading, especially for moving targets with larger bistatic delays. This behaviour limits the performance of the MC-FSR using OFDM waveforms as compared to the narrowband waveforms.

To counteract this effect, sub-band processing can be applied to reduce the effective bandwidth via pre-filtering, thereby slowing the decay of the autocorrelation function [6], while this approach improves the persistence of off-baseline target signatures, it also increases the correlation between the direct-path and target-path signals. This, in turn, may hinder the detection of stationary targets. To address this trade-off, spatial smoothing can be introduced to mitigate the increased correlation, thus enhancing the separability of stationary targets while retaining improved visibility for moving ones. Together, sub-band processing and spatial smoothing provide a complementary strategy for balancing detection performance in mixed-target scenarios, as both are governed by the autocorrelation characteristics of the waveform.

## 5. Simulation Results

This section presents simulation-based performance analysis of the MC-FSR using the proposed MUSIC-based (Figure 2) processing scheme while we specifically compare it to the FFT-based (Figure 1b) scheme. In our preliminary work [13], we showed the effectiveness of the MUSIC-based method with sinusoidal waveforms. In this paper, however, our focus is on more realistic scenarios using OFDM waveforms.

In the first set of analyses in this section (first two subsections), we focus on simplified single-target scenarios—either stationary or moving—to isolate and examine specific aspects of the proposed method’s performance as a function of the relevant parameters. In the second part, we consider a more challenging multi-target configuration involving both stationary and moving targets. This enables a comprehensive evaluation of the overall system performance and, in particular, a clear comparison with the FFT-based space-domain approach. The main parameters of the simulations are reported in Table 1.

### 5.1. Correlation Effect

**Figure 3:**
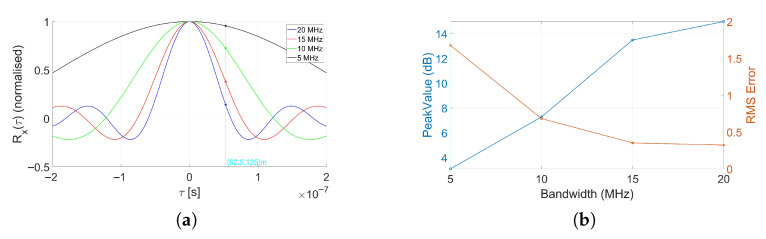
(**a**) Autocorrelation function of the transmitted waveform (bistatic delay corresponding to the target location is specified by a vertical line). (**b**) Peak value and angle estimation RMSE of the stationary target versus bandwidth (N=12).

To see the effect of signal autocorrelation on stationary targets, using an RX array with N=12 antennas, we consider a single stationary target of rectangular shape with dimensions (17 m × 3.6 m) resembling the size of a helicopter, located at (62.5,125), corresponding to a direction of arrival of 26.56° and a bistatic delay of $\tau_{\mathrm{bi}}=53\;\mathrm{ns}$. We examine the full-band case as well as sub-bands representing 3/4, 1/2, and 1/4 of the nominal bandwidth. Owing to the guard band included in the simulated OFDM signal, the resulting effective bandwidths become 16.7, 13.12, 8.12, and 3.12 MHz, respectively. The corresponding autocorrelation functions for these bandwidths are shown in Figure 3a, where the target delay is marked with a horizontal reference line. From Figure 3a, it is evident that reducing the bandwidth enhances the cross-correlation between the stationary-target return and the direct-path signal, while we could potentially expect a performance degradation of the proposed MUSIC-based method by an increase in correlation, as explained earlier in Section 4, there is also a competing effect inherent to the FSR principle: the desired signal amplitude is proportional to the waveform autocorrelation, which becomes larger as the bandwidth narrows. Furthermore, a third, less influential factor is the reduction in received signal power caused by sub-band filtering.

**Figure 4:**
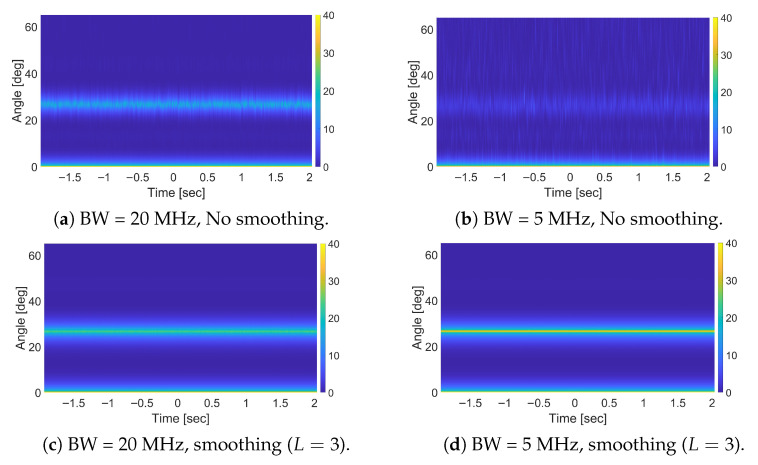
MUSIC-based method output map for a stationary target using different bandwidths, with and without spatial smoothing.

We apply the MUSIC-based approach in all bandwidth settings and evaluate performance in terms of (i) the peak value normalized with respect to the background level and (ii) the RMSE of the estimated angle. The corresponding curves are shown in Figure 3b, and the numerical values are summarized in Table 2. We have also included the maps obtained through illustrating the obtained pseudo-spectrum over an observation period for full-band and 1/4-band cases in Figure 4a,b. The results confirm our earlier discussion in Section 4, about the advantageous effect of signal bandwidth on the decorrelation between the direct-path and stationary-target components, which in turn improves the MUSIC performance. Such an improvement, which increases as the signal bandwidth widens, occurs in spite of the reduction in the received signal power due to the FSR principle. This further highlights the sensitivity of the proposed MUSIC-based method to the correlated components.

The results obtained after applying spatial smoothing are also reported in Table 2, and the obtained maps for full-band and 1/4-band cases are also shown in Figure 4c,d. As is evident, spatial smoothing consistently improves performance across all bandwidth settings, albeit at the cost of increased computational complexity. However, its relative effectiveness depends on the level of correlation between the direct-path and stationary-target components. With larger bandwidths, the waveform itself already provides substantial decorrelation, and therefore the marginal benefit of spatial smoothing is smaller. Moreover, in this case, the target forward scatter amplitude fading might become the limiting effect. In contrast, with narrower bandwidths—where correlation is more pronounced—spatial smoothing not only resolves the correlated-components issue, but also restores the inherent advantage of operating with reduced bandwidth, since the same bistatic delay is observed withing the autocorrelation main lobe and yields higher energy for the desired signal (see Figure 3a). Consequently, the optimal configuration when employing spatial smoothing is achieved with the 1/4-band sub-band.

### 5.2. Target Velocity Effect

**Figure 5:**
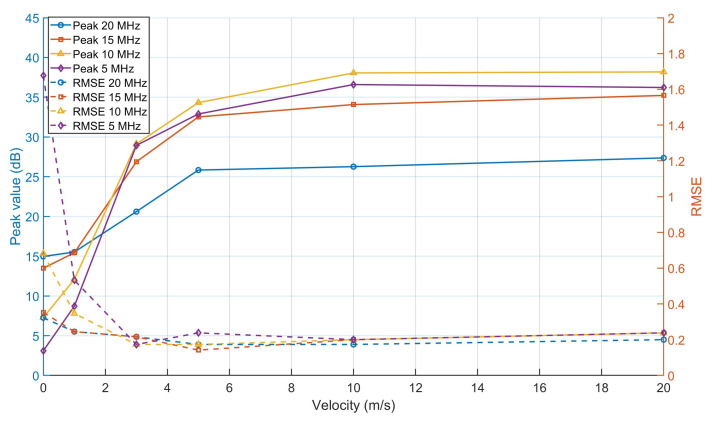
Peak value and angle estimation RMSE vs. target velocity for different bandwidth values (N=12).

**Figure 6:**
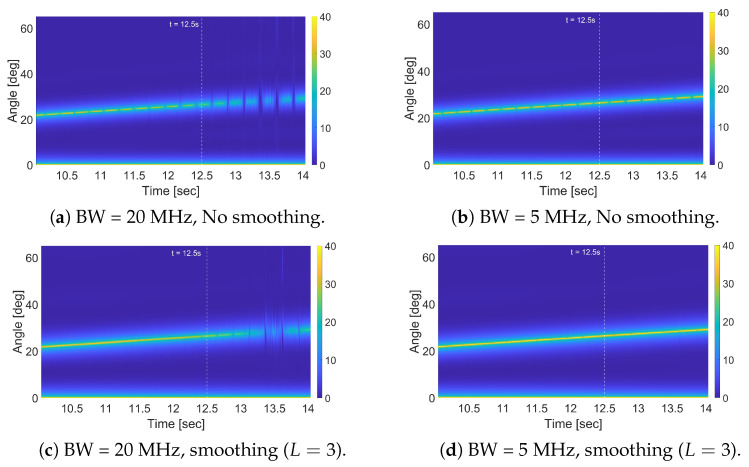
MUSIC-based method output map for a moving target (v=5 m/s) using different bandwidths, with and without spatial smoothing.

In this subsection, we repeat the analyses from the stationary-target case, but now using moving targets. For a fair comparison, each moving target is assigned the same physical dimensions as the stationary target. Further, each target moves along a trajectory orthogonal to the baseline and passes through the same reference point occupied by the stationary target in the previous tests, at the time of evaluation; at that moment, the MUSIC outputs are compared across different velocities. The results for different velocities, including zero-velocity case corresponding to the stationary target, are reported in Table 3 and the corresponding curves are shown in Figure 5. Similar results when using spatial smoothing are given in Table 4. We have also plotted the output maps for the case of v=5 m/s for BW=20 MHz and BW=5 MHz with and without spatial smoothing in Figure 6.

When no smoothing is applied, the performance consistently improves as target velocity increases (see Table 3) for any considered bandwidth setting. This behavior directly follows from the Doppler-induced time-varying phase ϕ(t), which decorrelates the direct-path and target components, as discussed in Section 4. It is also observed that the improvement saturates for sufficiently high velocities, consistent with our theoretical argument that, for fast-moving targets, $E[e^{j\phi (t)}]\to 0$, leading to a nearly complete decorrelation. In such cases, spatial smoothing offers limited additional benefit, as also confirmed by the numerical results in Table 4.

Another important observation from Table 3 concerns the role of bandwidth, while reducing the bandwidth was detrimental for stationary-target detection due to increased correlation, it becomes advantageous for moving targets. Once the correlated-components issue is mitigated by the Doppler-induced decorrelation, the dominant factor becomes the amplitude of the desired target response, which is proportional to the autocorrelation function. As the bandwidth narrows, the bistatic delay moves away from the autocorrelation null and toward its main lobe (see Figure 6), resulting in a higher target response despite the loss of some spectral energy from sub-band filtering.

Given these observations, we come again to the conclusion that a combined strategy of spatial smoothing and sub-band approach is the best compromise for realistic scenarios that we would expect both stationary and moving targets. This conclusion is fully supported by the results in Table 4, where the best overall performance for both target types occurs at the 1/2- and 1/4-band sub-bands.

### 5.3. Performance Evaluation in Multi-Target Scenarios

**Figure 7:**
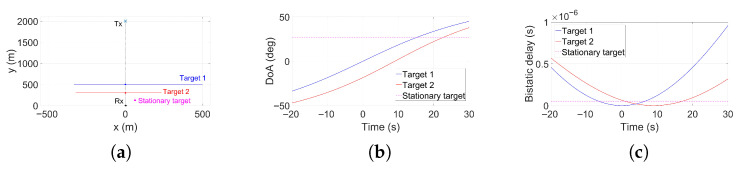
Ground-truth geometry: (**a**) target trajectories, (**b**) DoA vs. time, (**c**) bistatic delay vs. time.

The main simulation parameters are summarised in Table 1. An OFDM waveform with a bandwidth of 20 MHz is employed. The scenario consists of two moving targets and one stationary target, all modelled as rectangular objects. The corresponding target parameters, including size, velocity, and location, are listed in Table 5. To provide a clearer illustration of the overall geometry, the trajectories of the two moving targets and the position of the stationary target are shown in Figure 7a in local cartsian coordinates. Figure 7b,c report the corresponding DoAs and bistatic delays as functions of time, respectively. Finally, the multi-target configuration is evaluated using two array settings, with N=32 and N=12 receiving elements, in order to assess detection performance under both well-resolved and constrained array conditions.

**Figure 8:**
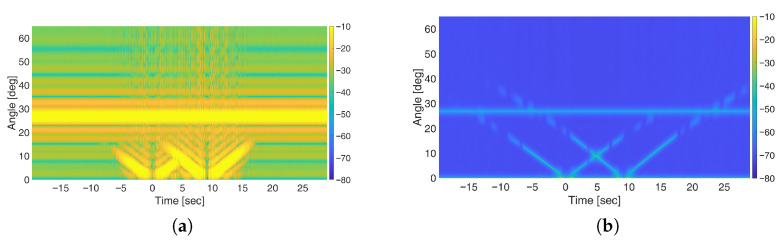
Results of simulations for the multi-target scenario using OFDM signals: (**a**) FFT-based space-domain processing; (**b**) MUSIC-based processing.

Figure 8 present the results obtained using FFT-based and the MUSIC-based methods, respectively, when using N=32 Antennas. As illustrated, the MUSIC-based method offers a significant improvement in angular resolution compared to FFT-based beamforming. Notably, the stationary target is also successfully detected, even in the absence of spatial smoothing thanks to the inherent properties of OFDM signals which help reducing the correlation between direct and target path signals when the full-band is retained. Actually, a partial correlation is still present since the stationary target is observed with amplitude level comparable with that of the moving targets, which are much smaller in size. However, such residual correlation does not prevent the detection of the target therefore spatial smoothing could be avoided thereby reducing the computational burden of the processing chain.

**Figure 9:**
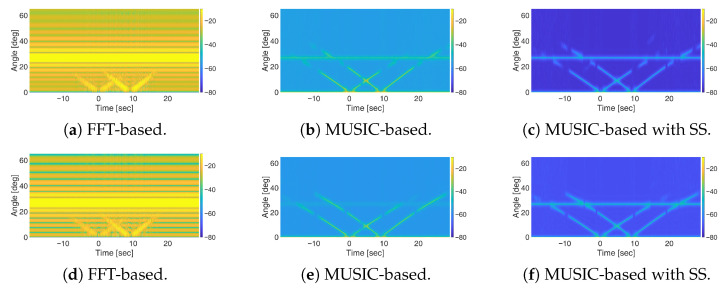
Results of simulations for OFDM waveform using (**a**–**c**) $\frac{1}{2}$-sub-band and (**d**–**f**) $\frac{1}{4}$-sub-band for different space-domain processing schemes: FFT-based, MUSIC-based, and MUSIC-based with spatial smoothing (SS) with L=3..

However, due to the use of the full available bandwidth of 20 MHz, while moving targets remain detectable, their visibility is limited to a short duration around the intersection point. Rapid attenuation of moving target signatures under OFDM waveforms is primarily attributed to the sinc-shaped autocorrelation function, which decays as the delay increases [6,12]. To address this issue, the sub-band processing approach is applied using sub-bands of one-half and one-fourth of the total bandwidth (see Figure 9). As evident, the sub-band approach has no impact on the FFT-based processing output, as the presence of a strong stationary target—observed at coarse resolution and full dynamic range—hinders the detectability of moving targets. In contrast, with the MUSIC-based approach, reducing the bandwidth from full (Figure 8b) to one-half (Figure 9b) and one-fourth sub-bands (Figure 9e) effectively mitigates the severe fading of the moving target responses, thus extending the time interval in which they can be easily discriminated from the background. However, this improvement comes at the cost of increased correlation between signal sources, which degrades the detectability of the stationary target. In such cases, applying spatial smoothing with a properly chosen number of subarrays *L* enables a balanced performance in preserving the visibility of stationary targets while enhancing the detection of moving ones, as shown in Figure 9f.

**Figure 10:**
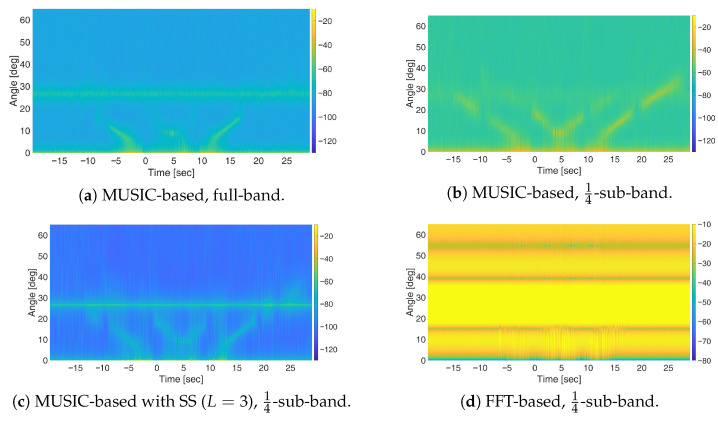
Results of simulations for OFDM waveform with *N* = 12: (**a**) MUSIC-based processing with fullband; (**b**) MUSIC-based processing with $\frac{1}{4}$-sub-band; (**c**) MUSIC-based processing with $\frac{1}{4}$-sub-band and spatial smoothing (*L* = 3); (**d**) FFT-based processing with $\frac{1}{4}$-sub-band.

We also examined a constrained scenario with only *N* = 12 receiving array elements, while keeping all other parameters unchanged. Figure 10a presents the results of MUSIC-based processing with fullband. It is clear that, with a reduced number of antennas, the detection of both stationary and moving targets becomes substantially more challenging. Applying the sub-band approach (Figure 10b) improves the visibility of moving targets over an extended duration but suppresses the stationary target response due to its increased correlation with the direct-path signal. In this case, combining the sub-band approach with spatial smoothing with L=3 (Figure 10c) enhances the stationary target response compared to the sub-band approach alone but this comes at the cost of reduced performance for moving targets. This behaviour highlights the increased sensitivity of the processing scheme to the loss of DoFs when only a limited number of antennas is available. It is worth noting that the FFT-based processing with sub-band approach (Figure 10d) fails to provide meaningful results in this multi-target scenario with just *N* = 12 antenna elements, further highlighting the effectiveness of MUSIC-based methods in such scenarios.

## 6. Experimental Tests

**Figure 11:**
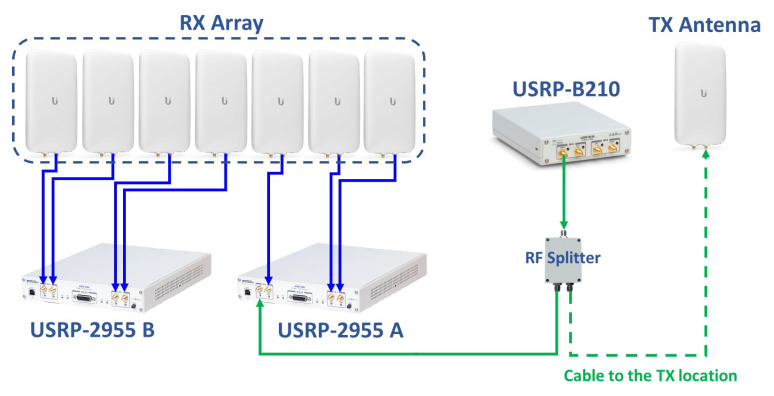
Representation of the acquisition setup.

To validate the performance of the proposed MUSIC-based MC-FSR framework using OFDM signals, an experimental campaign was carried out using the parameters reported in Table 6. The acquisition setup is illustrated in Figure 11. On the transmitter side, an Ettus USRP-B210 (Ettus Research, Austin, TX, USA) was used to generate either an OFDM signal at a carrier frequency of $f_{c}=2.477$ GHz and a bandwidth of B=20 MHz, controlled via MATLAB R2024b (MathWorks, Natick, MA, USA) The signal was split using an RF splitter: one branch fed the transmitting antenna, while the other was routed to the receiver circuitry for monitoring transmission activity. As the processing schemes employed in this study are reference-free, the monitoring signal was not used for signal processing. The receiver array was placed 37 metres away from the transmitter and consisted of a ULA with N=7 vertically polarised Ubiquiti UMA-D antennas (Ubiquiti Inc., New York, NY, USA), spaced at d=14 cm intervals. Reception was handled by two NI USRP-2955 devices National Instruments, Austin, TX, USA) (designated A and B), each offering four independent receiving channels. The received signals were individually down-converted, digitised, and forwarded to a host PC for offline processing. Although the two devices operated without a shared reference clock, thus lacking phase synchronisation, coarse time alignment and amplitude calibration were achieved using the direct-path signal from the transmitter.

**Figure 12:**
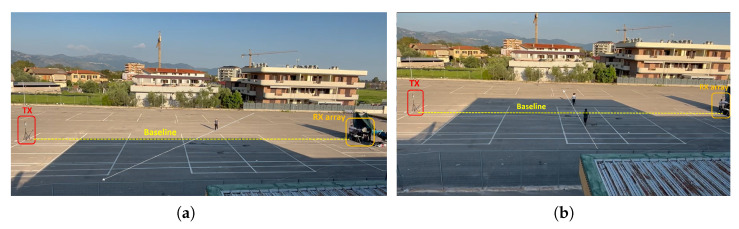
Geometry of the acquisition tests: (**a**) single target moving diagonally; (**b**) dual targets moving orthogonally.

Due to the chosen inter-element spacing (d≈1.156λ), angular ambiguities are expected, limiting the unambiguous detection sector to $u=\sin\left(\beta_{T}\right)\in \left[0,\;0.43\right]$, corresponding to an angular range of $\beta_{T}\in \left[0{}^{\circ},\;25.63{}^{\circ}\right]$. A number of experiments were performed to assess detection performance on human targets and drones moving along paths that intersect the baseline at its midpoint. A summary of test scenarios is provided in Table 7, and the corresponding geometrical configurations are depicted in Figure 12. It is worth noting that, given the limited range of bistatic delays in the adopted short-baseline geometry, target fading due to waveform autocorrelation is not expected. Therefore, the sub-band approach is not adopted in either of the tests, as it would not provide any significant advantage and would instead cause attenuation in the received signal power.

**Figure 13:**
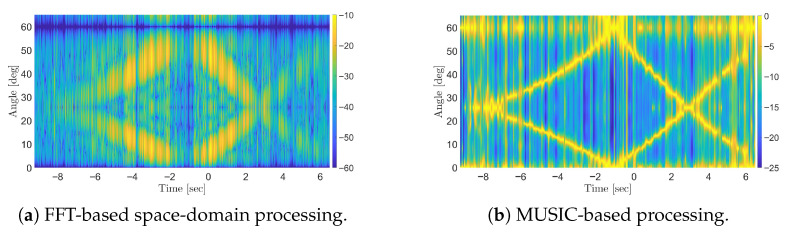
Experimental results for test 1.

In Test 1, a single moving target scenario is examined. We set p=3 to account for the moving target and the direct path signal and M=4N=28 samples were used for the estimation of the covariance matrix. The MC-FSR results obtained with FFT-based space-domain processing and the MUSIC-based processing are shown in Figure 13. Despite the strong multipath in the environment and inherent experimental uncertainties, both approaches successfully reveal the target’s presence through the characteristic V-shaped signature thus demonstrating the practical effectiveness of the considered amplitude-based sensing. However, the angular resolution and the clarity of the target signature achieved using MUSIC is substantially superior to that of FFT-based space-domain processing, underscoring its ability to resolve closely spaced features more precisely. These findings highlight the advantages of the MUSIC-based MC-FSR method, especially under hardware-constrained setups, such as those using a small RX array with N=7 elements. It is also important to note that insufficient spatial sampling, combined with the sign ambiguity, causes spectral folding effects that introduce spurious peaks in the angular domain. Notably, since no cancellation is performed in the MUSIC-based approach, the replica of the direct path signal appears approximately at 60° in the output map while there appears a null in the FFT-based output.

**Figure 14:**
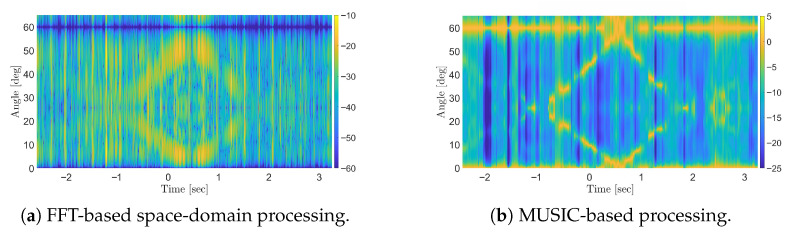
Experimental results for test 2.

To assess the system’s capability to detect small, weak targets, Test 2 was conducted using the same experimental parameters, with a small drone (DJI Mavic Pro) acting as the target. We set p=3 and M=28 for this test, too. As illustrated in Figure 14, the target signature is barely distinguishable with a MC-FSR using FFT-based beamforming due to its low signal power. In contrast, applying the MUSIC algorithm not only enhances angular resolution but also improves the target’s visibility against background clutter. These results further validate the effectiveness and superiority of MUSIC-based processing scheme for improving the detection of weak targets with MC-FSR, compared to the original FFT-based space-domain approach.

**Figure 15:**
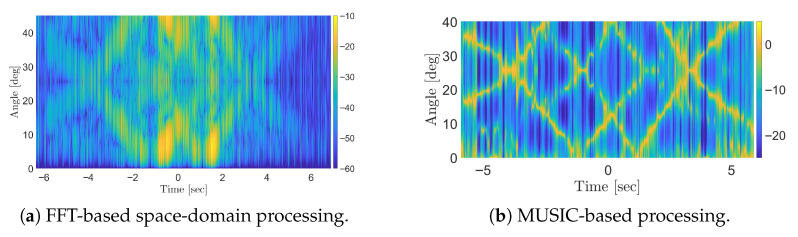
Experimental results for test 3.

To further evaluate the MUSIC-based approach performance in MC-FSR operated in multi-target scenarios, an experiment was conducted involving two human targets running (one after the other) and intersecting the baseline orthogonally (Figure 12b). All other processing parameters were kept the same, except for the assumed number of DoFs, which was set to p=5 to account for the two targets and the direct-path signal. The results, shown in Figure 15, reveal that the MC-FSR using the FFT-based space-domain processing scheme struggles to resolve the two targets, especially when their signatures intersect in the angle–time map. In contrast, the MC-FSR exploiting the MUSIC-based scheme successfully distinguishes between them, clearly demonstrating its superior performance and better angular resolution. Importantly, even under such constrained and borderline conditions with only N=7 RX elements and p=5 DoFs, the MUSIC algorithm maintains effective multi-target resolution. Note that here, given the use of an OFDM waveform in fullband (which was shown beneficial in decorrelating signals) and the limited available DoFs, spatial smoothing turns to be both unnecessary and impractical. These findings further support the earlier analysis that spatial smoothing is not strictly required when using OFDM in such conditions.

## 7. Conclusions

This paper presented a comprehensive framework for applying the MUSIC algorithm to MC-FSR systems using amplitude-only, real-valued data and arbitrary waveforms with a special focus on ubiquitous OFDM signals. The proposed approach addresses key challenges in FFT-based space-domain processing, such as limited angular resolution and difficulties in resolving weak or closely spaced targets, particularly when using small antenna arrays to keep the system low-cost. The impact of OFDM waveforms was analyzed, revealing that the inherent autocorrelation properties aid in decorrelating the direct and target-path signals, which can make spatial smoothing unnecessary even for stationary targets in fullband operation. A sub-band approach with spatial smoothing was proposed to balance enhancing moving-target visibility and preserving stationary-target detectability.

Simulation results confirmed the improved performance and target discrimination capabilities of the MUSIC-based approach over the FFT-based space-domain processing scheme. Further validation was carried out through experimental trials with a compact MC-FSR system using commercial hardware and WiFi-based waveforms. These real-world tests confirmed the superiority of the MUSIC-based scheme over FFT-based processing in terms of angular resolution and target visibility, particularly for weak targets (drone) and in multi-target scenarios. The findings support the potential of the proposed method for practical applications, particularly in emerging ISAC systems.

## Figures and Tables

**Table 1:** Parameters used for simulated results.

Parameter	Value
Signal frequency	$f_{c}=2.4$ GHz
First RX antenna coordinates	(0, 0) m
TX antenna coordinates	(0, 2000) m
Number of RX antennas	N=12,32
Array inter-element spacing	d=λ/2=0.0625 m
Array tilt angle	$\alpha_{s}=0$
DNR	40 dB
Waveform	OFDM
Nominal BW	20 MHz
Effective BW	16.7 MHz

**Table 2:** Performance of the MUSIC-based method with N=12 for stationary target using different bandwidth values, with and without spatial smoothing (L = 3).

		Bandwidth (MHz)			
		20	15	10	5
**No smoothing**	Peak Value (dB)	14.97	13.49	7.28	3.10
	DoA RMSE (deg)	0.32	0.35	0.68	1.68
**Spatial smoothing (L = 3)**	Peak Value (dB)	23.93	34.28	38.81	40.41
	DoA RMSE (deg)	0.19	0.19	0.11	0.10

**Table 3:** Performance of the MUSIC-based method with N=12 for targets moving at different velocities without smoothing.

No Smoothing		Velocity (m/s)				
		v=0	v=1	v=3	v=10	v=20
**20 MHz**	Peak value (dB)	14.97	15.52	20.61	26.27	27.37
	DoA RMSE (deg)	0.32	0.24	0.22	0.17	0.20
**15 MHz**	Peak value (dB)	13.49	15.43	26.88	34.08	35.23
	DoA RMSE (deg)	0.35	0.24	0.22	0.20	0.24
**10 MHz**	Peak value (dB)	7.28	12.08	29.12	38.06	38.19
	DoA RMSE (deg)	0.68	0.35	0.17	0.20	0.24
**5 MHz**	Peak value (dB)	3.10	8.71	28.94	36.60	36.23
	DoA RMSE (deg)	1.68	0.53	0.17	0.24	0.24

**Table 4:** Performance of the MUSIC-based method with N=12 for targets moving at different velocities and smoothing with L = 3.

Smoothing (L = 3)		Velocity (m/s)				
		v=0	v=1	v=3	v=10	v=20
**20 MHz**	Peak value (dB)	23.93 dB	25.41 dB	26.10 dB	28.37 dB	28.40 dB
	DoA RMSE (deg)	0.19	0.20	0.20	0.14	0.20
**15 MHz**	Peak value (dB)	34.28 dB	34.79 dB	34.68 dB	36.57 dB	36.86 dB
	DoA RMSE (deg)	0.19	0.20	0.20	0.20	0.24
**10 MHz**	Peak value (dB)	38.81 dB	40.43 dB	39.60 dB	41.70 dB	41.49 dB
	DoA RMSE (deg)	0.11	0.20	0.20	0.20	0.27
**5 MHz**	Peak value (dB)	40.41 dB	41.18 dB	38.80 dB	40.09 dB	40.38 dB
	DoA RMSE (deg)	0.10	0.20	0.20	0.24	0.30

**Table 5:** Parameters used in the multi-target scenario.

Parameter	Value
**Moving targets:**	
Size	Small UAVs (0.3 m × 0.4 m)
Velocities	$v_{1}=$ 60 km/h, $v_{2}=$ 40 km/h
Crossing point	$P_{1}=\left(0,500\right)$ m, $P_{2}=\left(0,300\right)$ m
**Stationary target:**	
Size	Helicopter (17m × 3.6m)
Location	(62.5 m, 125 m)
DoA of stationary target	26.5°

**Table 6:** Parameters used for experimental results.

Parameter	Value
Carrier Frequency	$f_{c}=2.477\;\mathrm{GHz}$
Direct signal to noise ratio	DNR≃20dB
Waveform type	OFDM with 16-QAM constellation
Bandwidth	B=20MHz
Number of RX Antennas	N=7
Inter-antenna spacing	d=14cm
RX location	(0,0)
TX location	(0,37) m
Array tilt angle	$\alpha_{s}=0{}^{\circ}$
Baseline length	B=37m

**Table 7:** Experimental tests information.

Test	Description
1	One human moving diagonally
2	One drone moving orthogonally
3	Two human running orthogonally

## Data Availability

Dataset available on request from the authors.
